# Chemical Sensors and Biosensors for Point-of-Care Testing of Pets: Opportunities for Individualized Diagnostics of Companion Animals

**DOI:** 10.1021/acssensors.4c03664

**Published:** 2025-04-22

**Authors:** Wilson Tiago Fonseca, Tatiana Parra Vello, Gabrielle Coelho Lelis, Ana Vitória Ferreira Deleigo, Regina Kiomi Takahira, Diego Stéfani Teodoro Martinez, Rafael Furlan de Oliveira

**Affiliations:** † Brazilian Nanotechnology National Laboratory (LNNano), 215006 Brazilian Center for Research in Energy and Materials (CNPEM), 13083-100 Campinas, Brazil; ‡ Mackenzie Institute for Research in Graphene and Nanotechnologies (MackGraphe), Mackenzie Presbyterian Institute (IPM), 01302-907 São Paulo, Brazil; § Institute of Chemistry (IQ), University of Campinas (UNICAMP), 13083-862 Campinas, Brazil; ∥ Post-Graduate Program in Materials Science and Technology (POSMAT), 28108 São Paulo State University “Júlio de Mesquita Filho” (UNESP), 17033-360 Bauru, Brazil; ⊥ Department of Veterinary Clinics, School of Veterinary Medicine and Animal Science, São Paulo State University “Júlio de Mesquita Filho” (UNESP), 18618-687 Botucatu, Brazil; # Institute of Physics “Gleb Wataghin” (IFGW), University of Campinas (UNICAMP), 13083-859 Campinas, Brazil

**Keywords:** point of care testing, lateral flow test, diagnostics, sensors and biosensors, companion animals, animal healthcare, veterinary diagnostics, one health

## Abstract

Point-of-care testing (POCT) is recognized as one of the most disruptive medical technologies for rapid and decentralized diagnostics. Successful commercial examples include portable glucose meters, pregnancy tests, and COVID-19 self-tests. However, compared to advancements in human healthcare, POCT technologies for companion animals (pets) remain significantly underdeveloped. This Review explores the latest advancements in pet POCT and examines the challenges and opportunities in the field for individualized diagnostics of cats and dogs. The most frequent diseases and their respective biomarkers in blood, urine, and saliva are discussed. We examine key strategies for developing the next-generation POCT devices by harnessing the potential of selective (bio)­receptors and high-performing transducers such as lateral flow tests and electrochemical (bio)­sensors. We also present the most recent research initiatives and the successful commercial pet POCT technologies. We discuss future trends in the field, such the role of biomarker discovery and development of wearable, implantable, and breath sensors. We believe that advancing pet POCT technologies benefits not only animals but also humans and the environment, supporting the One Health approach.

## Graphical abstract

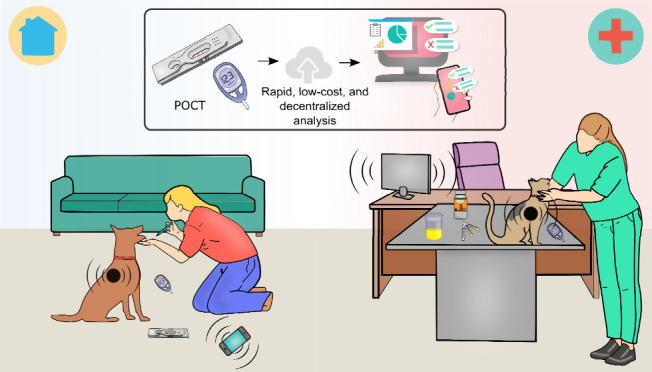

Throughout all human history, few relationships have been more successful than that between humans and their companion animals (pets).^1^ Since the appearance of the first domesticated wolves more than 12,000 years ago, pets (mostly dogs and cats) have been an integral part of modern human social and economic life.^1^ They provide not only companionship, but also serve in protection, security, hunting, herding, and guiding people with disabilities.^2^ Pets have also been reported to have positive effects on human physical and psychological health^3^ by reducing blood pressure and stress,^4^ in addition to amending loneliness, depression, and dementia.^5^ All these benefits have led to the increasing popularity of pets: around 87 million (or 63%) of US households own a companion animal according to the American Pet Products Association (APPA),^6^ an increase of 10% compared to three decades ago.^7^

The growing intimate relationship between humans and pets has also led to the so-called “pet humanization”, where companion animals are treated with extra level of care and attention.^8^ A recent survey found that 97% of pet owners consider their animals part of the family,^9^ while others view their pets as a form of “extended self”.^10^ This trend has significantly heightened concerns for pet health and well-being,^9^ driving increased investments in veterinary medicine and diagnostics.^11^ As animals receive extra care, nutrition, and medical attention, they live longer and develop age-related illnesses like arthritis, renal disease, and cancer.^8^ Their humanized lifestyle has also led to the development of diabetes and hypertension.^12^ Additionally, pets can transmit many diseases to humans (viz. zoonotic diseases) and be infected by humans (e.g., COVID-19).^13^ Thus, investing in pet diagnostics and health monitoring is beneficial to both animals and humans, ultimately enhancing the overall well-being of our society.

Point-of-care testing (POCT) plays an important role in medical diagnostics when rapid, low-cost, and decentralized analysis is required.^14,15^ In veterinary medicine, POCTs are particularly vital because animals cannot verbally communicate their symptoms easily like humans, making early illness detection more challenging.^15^ As a result, diseases often progress unnoticed, leading to delayed intervention. In addition, POCT supports individualized pet healthcare, where therapies are tailored to the specific needs of each animal.^16^ Thus, developing POCT for pets enables prompt diagnosis and treatment, reducing overall veterinary costs. (Figure 1).

**Figure 1:**
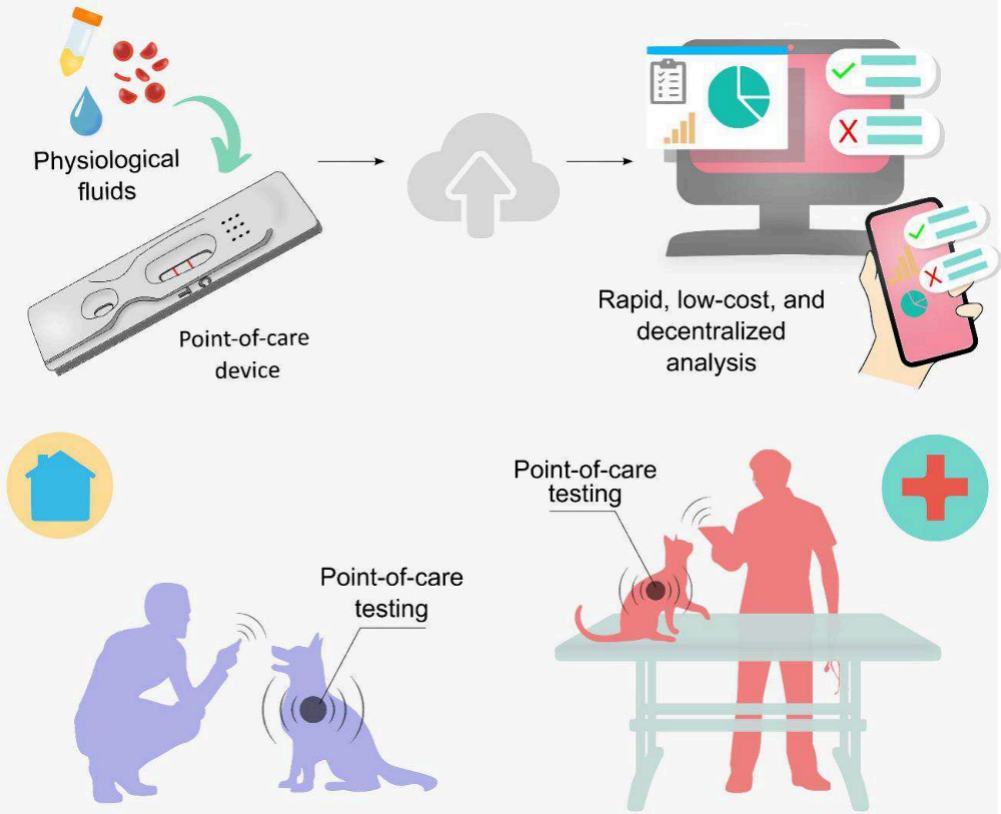
Schematic representation of POCT for diagnosis of pets. Portable devices for detecting biomarkers in animal fluid aim for rapid, low-cost, and decentralized analysis. Information can be stored in cloud servers or transmitted to hand-held devices for domestic health monitoring or veterinary care.

Another positive aspect of advancing POCT for pet diagnostics and health monitoring is its economic potential and market opportunity. The predictions for the POCT market in pet diagnostics are highly promising, with a projected growth of USD 4.1 billion by 2032.^17^ In comparison, the POCT market for human diagnosis is currently worth 16 times more than that for pets (USD 38.6 billion in 2022).^18^ Such a thriving market is driven by continuous and extensive efforts in research and innovation. However, compared to the developments in human health, research initiatives in POCT for pets are significantly scarcer.

This review aims to comprehensively explore the current development of POCT for pet diagnostics, emphasizing research opportunities and strategies to address the most important challenges in the field. We begin by summarizing the most critical disease biomarkers in pets and their clinically relevant levels in physiological fluids (viz. blood, urine, and saliva). We focus on cats and dogs as they are the most prevalent companion animals in households.^7^ We discuss fluid sampling and handling practices, detailing their respective advantages and disadvantages for pet POCT. We explore key strategies for advancing the next-generation POCT devices by harnessing the potential of selective (bio)­receptors and high-performing transducers such as lateral flow tests and electrochemical (bio)­sensors. We also present the most recent research initiatives in the field and successful and commercially available POCT technologies. Finally, we discuss future trends and the requirements for enabling new sensing technologies toward individualized and improved pet diagnostics. We believe that developing POCT for pets can significantly benefit society, ultimately contributing to achieving a genuine human-animal-environmental One Health.^7^

## Biomarkers in Animal Fluids and Sample Collection

Biomarkers are biomolecules that can indicate normal or abnormal biological processes, diseases, and responses to treatment. They are classified into seven categories,^20^ although a single biomarker can belong to more than one classification. These include, (i) susceptibility biomarkers, which indicate the potential for development of diseases with no apparent symptoms (e.g., cancer biomarkers), (ii) diagnostic biomarkers, to identify patients with a certain disease, (iii) monitoring biomarkers, used to verify a change in degree or extent of disease, (iv) predictive biomarkers, to identify genetic predisposition to develop a particular disease, (v) prognostic biomarkers, to identify the possibility of disease recurrence or progression, (vi) pharmacodynamic or response biomarkers, to confirm whether a biological response was generated after administration of drugs or vaccination, and (vii) safety biomarkers, used to identify if the animal has been exposed to harmful environments.

Just like in humans, physiological fluids are a rich source of biochemical information, providing critical insights into the body’s health. The most common fluids for POCT of companion animals are blood, saliva, and urine due to the facile sample collection.^21^ Each fluid exhibits a large variety of biomarkers at different concentrations, providing unique (bio)­chemical information for comprehensive diagnostics, prognostics, and health monitoring^22^ (Figure 2). Identifying the target biomarker is a crucial step in the early stages of POCT development, affecting subsequent decisions like the choice of recognition element and the transducing technology. Tables 1 and 2 show the most common biomarkers in blood, urine, and saliva, their clinically relevant concentration and respective research efforts for dogs and cats.

**Figure 2:**
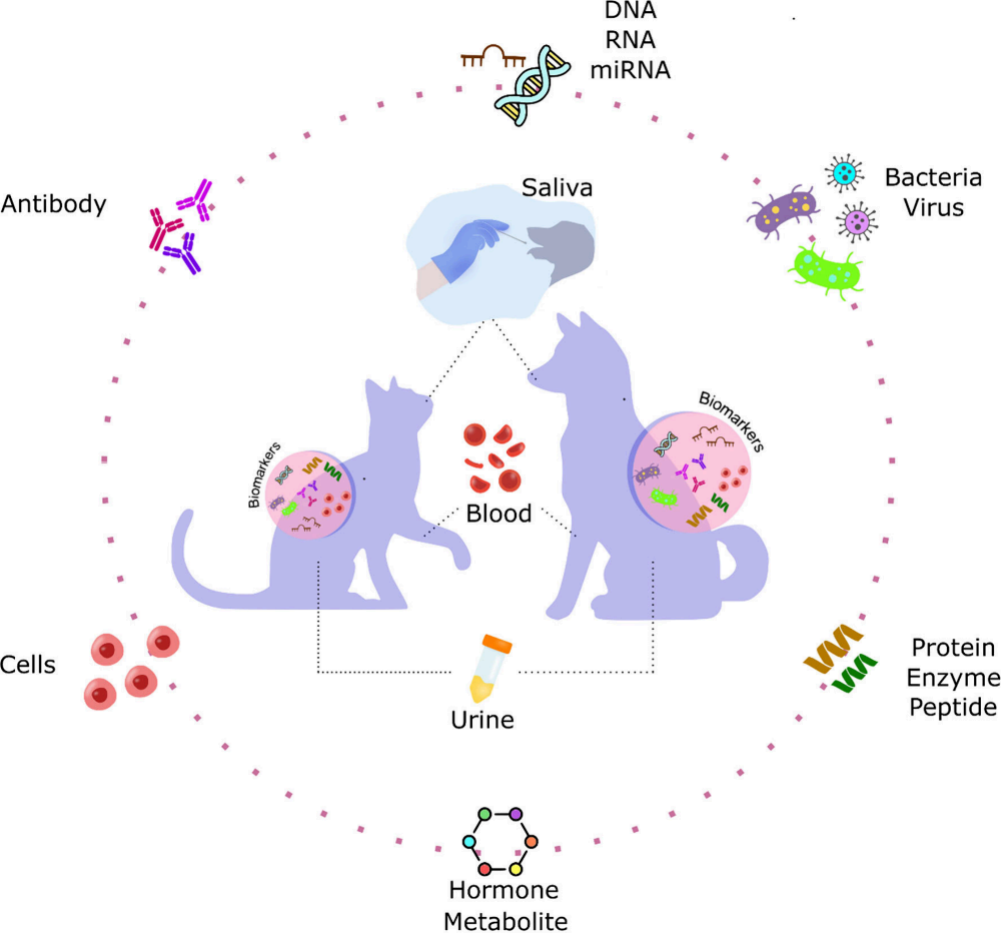
Main physiological fluids and types of biomarkers for disease diagnosis and health monitoring in dogs and cats.

**Table 1:** Most Common Canine Biomarkers and Respective Research Efforts^a^

types of biomarkers	target biomarker	fluid	disease	level	method	POCT	ref
hormone	GH	blood	acromegaly	≥10 ng/mL	RIA	no	^92^
hormone	cortisol	blood	AD	<2 μg/dL	ACTH test	no	^93^
hormone	AMH	blood	SCT	>22 ng/mL	ELISA	no	^94^
DNA	BC-DNA	blood	brucellosis		ELISA	no	^95^
DNA	Babesia DNA	blood	canine babesiosis		PCR	no	^96^
DNA	Leishmania infantum DNA	blood	LSH	20–2000 parasites/mL	RT-qPCR	no	^97^
RNA	CDV-RNA	blood	distemper	6.26–758 × 10^4^ RNA copies/μ	RT-qPCR	no	^98^
		urine		2.35 × 10^9^ RNA copies/μL			
miRNA	miRNA-214 and miRNA-126	blood	neoplastic disease		RT-qPCR	no	^99^
protein	CEA	blood	breast cancer	>0.23 ng/mL	RIA	no	^100^
protein	CRP	blood	infectious diseases (e.g., heartworm infection)	14.5–29.8 mg/L	ITD	no	^101^
protein	CRP	blood	infectious diseases (e.g., ehrlichiosis)	217.8–788.8 μg/mL	ELISA	no	^102^
protein	CPSE	blood	PH	≥61 ng/mL	ELISA	no	^103^
peptide	NT-pBNP	blood	heart failure	>1400 pmol/L	ELISA	no	^104^
peptide	ET1	blood	IPF	>1.8 pg/mL	ELISA	no	^105^
enzyme	ALT	blood	hepatitis	>1000 U/L	biochemical	no	^106^
enzyme	ADA	blood	pyometra	2.9–7.9 IU/L	biochemistry	no	^107^
		saliva		2.2–15.5 IU/L			
metabolite	creatinine	blood	CKD	>1.4 mg/dL	spectro	no	^108^
metabolite	urea	blood	azotemia	11.2–37 mmol/L	CCA	no	^75^
		saliva		3–11 mmol/L	SUTS	yes	
carb	glucose	blood	DM	>120 mg/dL	PBGM	yes	^74^
DNA	CAV-1	urine	ICH		PCR	no	^109^
bacteria	Lspp	urine	Lepto		IMS-PCR	no	^110^
protein	CAPG	urine	TCC		LC-MS/MS	no	^111^
peptide	Col2CTx	urine	OA	46.3–68.2 ng/mg Cr	ELISA	no	^112^
metabolite	UA	urine	HUU	501–597 mg/L	electrophoresis	no	^113^
protein	RVA	saliva	rabies		LAT	no	^114^
miRNA	miR-21	saliva	MCTs		RT–qPCR	no	^115^
metabolite	MDA	saliva	PD	36–833 ng/mL	ELISA	no	^116^

a GH: growth hormone, RIA: radioimmunoassay, AD: Addison’s disease, ACTH: Adrenocorticotropic hormone, AMH: Anti-Müllerian hormone, SCT: Sertoli cell tumors, ELISA: enzyme-linked immunosorbent assay, BC-DNA: *Brucella canis* DNA, PCR: polymerase chain reaction, LSH: Leishmaniasis, RT-qPCR: real-time quantitative PCR, CDV-RNA: canine distemper virus RNA, CEA: carcinoembryonic antigen, CRP: C-reactive protein, ITD: immunoturbidimetric, CPSE: canine-prostate specific arginine esterase, PH: prostatic hyperplasia, NT-pBNP: amino terminal-pro-B-type natriuretic peptide, ET1: endothelin-1, IPF: idiopathic pulmonary fibrosis, ALT: alanine aminotransferase, ADA: adenosine deaminase, CKD: chronic kidney disease, Spectro: spectrophotometric, CCA: clinical chemistry analyzer, carb: carbohydrate, DM: diabetes mellitus, PBGM: portable blood glucose meter, CAV-1: canine adenovirus-1, ICH: infectious canine hepatitis, Lspp: *Leptospira* spp, Lepto: leptospirosis, IMS-PCR: immunomagnetic separation technique with PCR, CAPG: macrophage capping protein, TCC: transitional cell carcinoma, LC-MS/MS: liquid chromatography tandem mass spectrometry, OA: osteoarthritis, UA: uric acid, HUU: hyperuricosuria, RVA: rabies virus antigen, LAT: latex agglutination test, MCT: mast cell tumors, MDA: malondialdehyde, PD: periodontal disease, SUTS: saliva urea test strip.

**Table 2:** Most Common Feline Biomarkers and Respective Research Efforts^a^

types of biomarkers	target biomarker	fluid	disease	level	method	POCT	ref
hormone	IGF–1	blood	acromegaly	>1000 ng	RIA	no	^117^
hormone	T4	blood	HT	>30 nmol/L	RIA	no	^118^
mRNA	FCoV RNA	blood	FCoV		RT–PCR	no	^119^
antibody	anti–LI	blood	LSH	>80 titer	IFAT	no	^120^
protein	SAA	blood	Infectious Diseases (e.g., FIP)	38.00–141.80 mg/L	ITD	no	^121^
protein	NGF	blood	BC	2.10–11.09 ng/mL	ELISA	no	^122^
enzyme	ALT	blood	ANC	12–2685 U/L	biochemical	no	^123^
enzyme	Feline TK1	blood	lymphoma	1.50–13.3^b^	spectro	no	^124^
metabolite	creatinine	blood	CKD	>1.6 mg/dL	spectro	no	^108^
		urine		UPC ≥ 0.2	colorimetric		^125^
metabolite	Urea	blood	azotemia	13.2–89.3 mmol/L	CCA	no	^75^
		saliva		3–8 mmol/L	SUTS	yes	
carb	glucose	blood	DM	>150 mg/dL	PBGM	yes	^74^
cell	leukocyte	blood	leukopenia	50–7000 cells/μL	Haema	no	^126^
miRNA	miR-21a^c^	urine	KD	50–10 000 amol/g/dL^c^	qPCR	no	^127^
DNA	Leptospira DNA	urine	Lepto	393–15 760 leptospires/mL	RT–qPCR	no	^128^
antibody	Anti-FIV	saliva	FIV		LFIA	yes	^77^
antibody	Anti-IgA	saliva	FSI	>11.5 units/mL	ELISA	no	^129^
protein	FeLV p27	saliva	FeLV infection		ELISA	no	^130^

a IGF-1: insulin-like growth factor 1, T4: thyroxine, HT: hyperthyroidism, mRNA: mRNA, FCoV: feline coronavirus, RT-PCR: reverse transcriptase PCR, anti-LI: anti-*Leishmania infantum*, IFAT: immunofluorescent antibody test, SAA: serum amyloid A, FIP: feline infectious peritonitis, NGF: nerve growth factor, BC: bacterial cystitis, ALT: alanine aminotransferase, ANC: acute neutrophilic cholangitis, TK1: thymidine kinase 1, Haema: hematological, KD: kidney disease, UPC: urine protein-to-creatinine ratio, FIV: feline immunodeficiency virus, LFIA: lateral flow immunochromatographic assay, IgA: immunoglobulin A, FSI: food sensitivities and intolerances, FeLV: feline leukemia virus.

b Results are expressed as TK1 activities in pmol/min/mL.

c miR-21a levels normalized with respect to urinary creatinine.

Blood is by far the most exploited physiological fluid in pet diagnosis and presents a wide range of biomarkers of infectious, metabolic, and endocrine diseases.^23^ In dogs, blood sampling is typically performed from the cephalic vein (in the foreleg), jugular vein (in the neck), or from the saphenous vein (in the hind leg),^24^ while in cats blood collection from cephalic or jugular veins is preferred.^25^ Blood can also be collected from the paw pad of cats and dogs. A major challenge in POCT for blood analysis is the variability in sample volume across different species and collection sites. Factors such as blood flow, skin thickness, and stress-induced resistance can complicate collection.^24,25^ Warming the collection site can improve blood flow and reduce discomfort. Gentle handling, optimizing animal position, and minimizing restrain time facilitate sampling. POCT devices typically require 0.3 μL of blood. To ensure accurate results, samples should be used immediately while fresh, as delays may lead to biochemical changes.

Blood analysis is invasive and sample collection can be uncomfortable for the animal, making urine and saliva interesting alternatives.^26^ Urine is the main physiological fluid for diagnosing renal^27^ and metabolic disorders.^28^ One of the main challenges of urine POCT in pets is sample collection and variability. Urine collection in dogs and cats is performed using the midstream method (free-catch collection), urethral catheterization, or cystocentesis.^29,30^ Free-catch collection involves the manual sampling of urine samples from voluntary urination, while urethral catheterization collects the urine directly from the urethra and cystocentesis involves extracting urine from the bladder.^30^ Free-catch can be carried out by nonprofessionals (e.g., pet owner), while catheterization and cystocentesis must be performed by a veterinarian to ensure high-quality samples and the proper handling of the animal.

Voluntary urination can exhibit significant volume variation depending on species and animal size. It is also highly prone to contamination (e.g., bacteria or environmental contaminants).^31^ Conversely, urethral catheterization and cystocentesis provide more control over sample collection but are invasive and stressful for the animal. Urine production depends on the animal hydration levels and recent activity levels.^32^ Typically, a few hundred of μL are necessary for POCT. Color inspection can help assess sample integrity, identifying potential degradation and contamination.

Saliva is also a very interesting noninvasive alternative for diagnosis and health monitoring.^33^ It has become more popular since the COVID-19 pandemic, where self-testing kits were commercialized in drugstores and used massively by the population. For animal diagnostics, saliva collection is faster, simpler, and less stressful compared to blood and urine sampling. Saliva collection is typically performed using a cotton swab or a specific mouthguard.^34^ Saliva collection can be easily carried out by non-experts such as pet owners, dog trainers, and cat sitters, facilitating the monitoring of the pet’s health. Saliva is a particularly interesting fluid for diagnosing viral and bacterial infections,^35^ in addition to periodontal diseases.^36^

A key challenge in saliva POCT for pets is ensuring reliable and consistent sample collection. Saliva composition and volume vary significantly based on species, breed, diet, and collection method.^34^ Swab-based sampling typically requires rubbing the swab inside the animal’s mouth for 30–60 s. Proper handling is essential to prevent insufficient sample collection or dilution errors. Once collected, the swab is transferred to a buffer solution to release the sample for analysis. If saliva volume is insufficient, stimulants such as citric acid or food scent can be used, although they can alter saliva composition. The sampling location within the mouth is reported to influence results depending on the analyte being measured.^34^ In dogs, allowing the animal to chew the collection device can enhance saliva production.^34^ The presence of the owner and sampling in an unfamiliar environment can also affect salivation. Finally, saliva viscosity and food residues can impact test accuracy, and samples visually contaminated with blood should be discarded.

Feces are also an important source of information about animal health, providing insights into gastrointestinal function, parasite infections, and microbiome composition. However, their use in POCT presents significant challenges. Sample collection is often inconsistent, particularly for animals that defecate outdoors or in uncontrolled environments, leading to variability in sample volume and freshness. Fecal samples also need a series of laborious pretreatments (e.g., dilution, homogenization and filtration), requiring immediate processing or preservation techniques. Improper sample preservation can result in bacterial proliferation or biomarker degradation, compromising diagnostic accuracy.^37^ Additionally, the lack of validated species-specific tests and the inherent variability of the fecal samples further reduce reliability compared to blood, urine, and saliva.

## POCT Technologies for Pets

To meet the rigorous standards for accurate and reliable diagnostics, POCT technologies must be designed to exhibit high sensitivity and selectivity, low limit of detection (LoD), reproducibility, robustness, rapid response, and affordability. One strategy is to adapt existing, validated human health technologies, e.g. commercial glucose meters, to the specific needs of pets. This approach can significantly reduce development costs and time while facilitating regulatory approval after animal clinical trials. However, human-designed devices may not perform optimally in pets due to physiological differences and adapting human POCT technologies can limit customization for individualized pet diagnostics. Alternatively, researchers can develop new sensing devices tailored to the specific needs of pets. In the following, we discuss the most common device platforms for POCT and strategies one can exploit to develop new sensing technologies for pets.

### Lateral Flow Tests (LFTs)

One of the most successful commercial platforms for POCT is the so-called lateral flow tests (LFTs) or immunochromatography assay (Figure 3). They consist of a functional paper strip encased in a small, portable plastic container aimed at detecting specific biomarkers in physiological samples (e.g., saliva, urine, nasopharyngeal secretion, blood, etc.).^38^ The sample collection and handling protocol (e.g., the use of swabs, buffers, the incubation time, etc.) may vary depending on the POCT technology and target disease (Figure 3a).^39^ LFTs can deliver fast results (a few minutes) at affordable prices, helping to screen patients for a potential disease without requiring traditional laboratory analysis performed by trained personnel.

**Figure 3:**
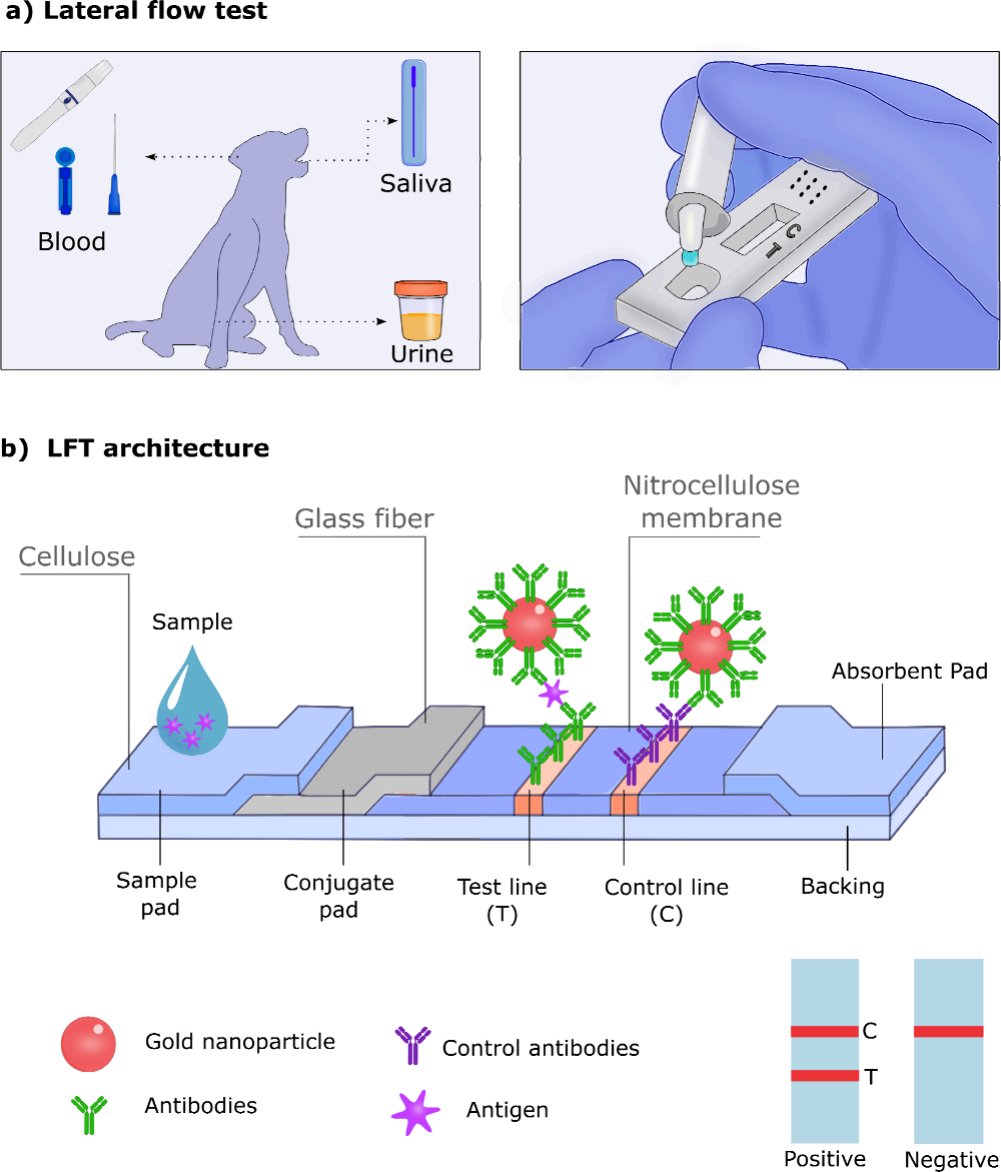
LFT device for biomarker detection and health monitoring. (a) LFT device and sample collection methods. (b) LFT architecture and components. Adapted and reproduced from ref 39. Copyright 2023 Springer Nature Limited.

A typical LFT features an inlet port for sample addition and a detection window.^40^ Internally, a paper strip comprises a sample pad that filters interfering species and transports the sample to the conjugate pad via capillary action. In the conjugated pad, rehydrated tagged receptors, typically Au nanoparticles (AuNPs)-labeled antibodies (Ab) bind to the target analyte (e.g., antigen or Ab), forming complexes that flow to the test line, where immobilized receptors capture them, causing a color change if the target is present. The control line captures excess tagged Ab, validating the sample flow and the device functionality (Figure 3b). An absorbent pad at the very end of the device collects the sample excess. Positive results show two colored lines, while negative results display one.

LFTs are valued for their affordability, ease of use, and rapid response time (≤1000 s),^41^ enabling access to diagnostics and health monitoring for pet owners and veterinarians, at home, in private clinics and public hospitals. They are used in veterinary medicine mainly for detecting infectious diseases (e.g., canine parvovirus, feline immunodeficiency virus - FIV, etc.) and metabolic markers (e.g., glucose). Their ability to provide rapid, on-site results accelerates decision-making, facilitating timely intervention – particularly for managing infectious and zoonotic diseases.

Despite their advantages, LTFs have limitations that prevent unrestricted use in definitive diagnostics. The method is generally not quantitative, limiting its application in cases requiring precise biomarker detection and quantification.^41^ Additionally, LFTs exhibit significant variability between devices and a high occurrence of false-positive and false-negative results.^41^ Moreover, since LFTs are commonly used as “do-it-yourself” tests, the absence of clear guidelines for interpreting positive results can lead to misdiagnosis and inappropriate clinical decisions.

### Materials and Strategies for LFTs

Advancements in LFTs for pet diagnostics should focus on improving sensitivity and reliability to ensure accurate results. Significant efforts have been devoted to optimizing materials to enhance the LFT performance.^42^ For example, the sample pad must exhibit high liquid absorption and release, while also functioning as a filter to remove interfering species. During sample addition, it should accommodate large sample volumes (tens of μL/cm^2^) and ensure an even and controlled distribution to the conjugate pad.^43^ The sample pad is also used to load (bio)­chemicals (e.g., proteins, surfactants, viscosity enhancers, etc.) often necessary to modulate the sample flow. Cellulose fibers are preferred for their high bed volume, cost efficiency, and ability to regulate fluid dynamics, serving as both sample and absorbent pads. The absorbent pad prevents sample overflow and backflow. Thus, selecting appropriate pad materials is essential for maintaining consistent flow and optimizing LFT performance.

The conjugate pad is responsible for delivering the sample and labeled recognition elements (e.g., tagged Ab) to the test membrane.^43^ Stored receptors rehydrate upon contact with the sample, bind to the target analyte, and are subsequently released. Thus, the conjugated pad must be porous, minimize nonspecific binding, and protect conjugates from denaturation while allowing controlled release. Glass fibers are preferred for their low protein affinity, while cellulose and surface-modified plastic fibers (e.g., polyester, polypropylene, polyethylene) can also be used.

The test membrane plays a crucial role in the capillary-driven analyte delivery to the detection zone, influencing response time, sensitivity, and signal intensity. Pore size regulates sample flow and reaction time, affecting signal strength.^43^ Nitrocellulose is the most commonly used membrane material due to its optimal capillary action, flow control, and high protein-binding capacity. The membrane is also used to accommodate the test and control lines, where the capture molecules are immobilized through a variety of chemical interactions.^43^ A polyethylene terephthalate substrate under the membrane provides structural support.

LFT sensitivity and accuracy strongly depend on the signal generation method. Most LFTs use colorimetric indicators, typically AuNPs, to produce visible detectable lines. However, low analyte concentrations may yield faint signals, making detection challenging.^44^ To enhance sensitivity and enable quantitative analysis, electronic readers (e.g., smartphone cameras) and advanced techniques, such as up-conversion nanoparticles, surface-enhanced Raman scattering and thermal contrast readers, have been explored.^45^ Additionally, multiplexing strategies, i.e. the ability to simultaneously detect multiple analytes in a single test or sample, include the incorporation of multiple recognition elements, tags, and test lines. For pet diagnostics, multiplexing is particularly useful for differentiating viral and bacterial infections, and for simultaneously assessing multiple health markers (e.g., inflammation, kidney function). Multiplexing enhances accuracy and improves diagnostics by providing a broader assessment of the pet’s health status during a single test.

Selecting appropriate bioreceptors is critical for LFT design, as they determine specificity, sensitivity, and overall performance. Bioreceptors are immobilized at both the test and control lines, and on detection tags (e.g., AuNPs). Ab are the preferred choice for detecting proteins, viral and bacterial antigens, and small molecules (e.g., hormones) due to their high affinity.^45^ However, high-quality Abs are expensive, very sensitive to temperature and pH changes, and may exhibit batch-to-batch variability.^45^ Abs also require oriented immobilization for more efficient performance. Thus, alternative bioreceptors have been considered for the next-generation LFTs, such as nanobodies, aptamers, peptide nucleic acid (PNA), DNA, and molecular beacons^46^ (Figure 4).

**Figure 4:**
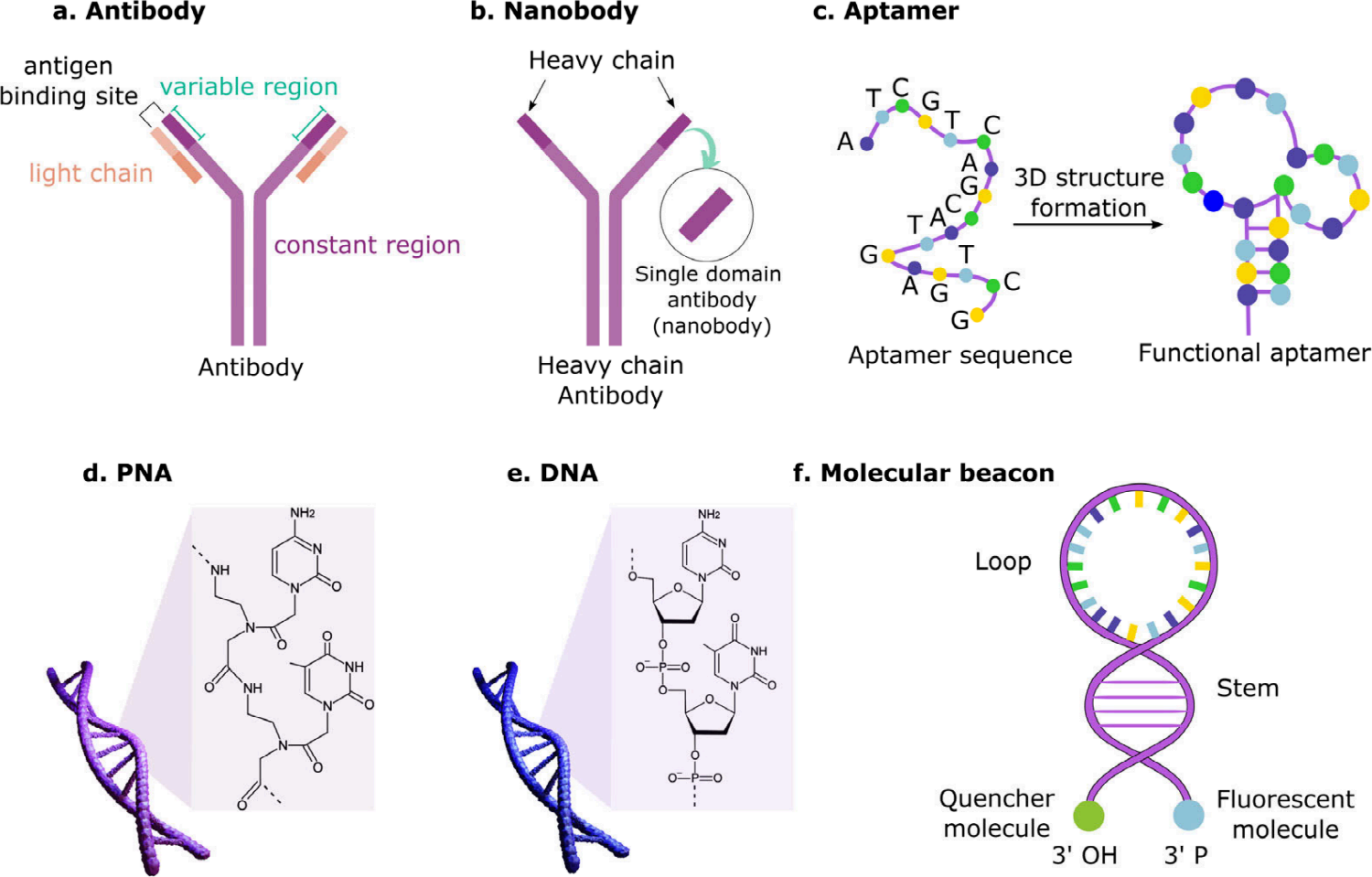
Bioreceptors for LFTs: (a) antibodies, (b) nanobodies, (c) aptamers, (d) PNA, (e) DNA, and (f) molecular beacons.

Nanobodies are single-domain Ab fragments with superior stability and solubility while maintaining high affinity and specificity.^47^ However, they are significantly smaller (15 kDa) than conventional Abs, making their immobilization challenging. Aptamers are interesting alternatives to overcome the limitations of Abs and nanobodies.^48^ They are single-stranded DNA (ssDNA) or RNA molecules that can be synthesized to recognize a wide range of analytes with high binding affinities.^48^ Upon interaction with the target analyte, they fold into unique three-dimensional structures, working as a molecular switch. They are easier and cheaper to produce and do not require oriented immobilization for efficient binding. Aptamers also display superior stability; however, their slower binding kinetics can compromise the LFT response time.^49^

PNA provides high specificity for complementary nucleic acid sequences associated with viral infections, cancer, and genetic disorders.^50^ PNA offer superior chemical and enzymatic stability and better capability to recognize single-base mismatches compared to DNA.^50^ However, DNA remains more widely used due to its lower cost and established protocols.^43^ Alternatively, molecular beacons, i.e., single-stranded DNA with a fluorescent probe and quencher can detect specific nucleic acids.^46^ Upon binding, molecular beacons undergo a conformational change that separates the quencher from the probe, emitting light.^46^ While there are ongoing research initiatives on PNA and molecular beacons as bioreceptors for diagnostics, no commercial LFTs exist using these strategies, especially for pets. For further information on LFT technology, readers are encouraged to refer to excellent review papers on the topic.^45,51^

### Electrochemical (Bio)­Sensors

Electrochemical sensors and biosensors represent another important class of transducers for POCT technologies.^52^ Portable glucose meters and coagulation tests are some successful examples of commercial devices used in both human and veterinary medicine. Electrochemical (bio)­sensors convert target analyte information, viz. presence and/or concentration, into a measurable electrical signal through a biorecognition process. Chemical receptors and (bio)­receptors, such as enzymes, Abs, aptamers, etc., immobilized on an electrode surface selectively interact with the analyte, and detection methods like amperometry, voltammetry, potentiometry and electrochemical impedance spectroscopy are used to generate and analyze the signal (Figure 5).^52^ Electrochemical POCT (bio)­sensors are mainly based on a miniaturized set of working (WE), reference (RE), and counter (CE) electrodes. A drop of sample, such as blood or other bodily fluid, is applied to the sensor surface and the integrated electronics record and converts the signal into a readable output.^53^ Typical detection methods are amperometry, voltammetry, and electrochemical impedance.

**Figure 5:**
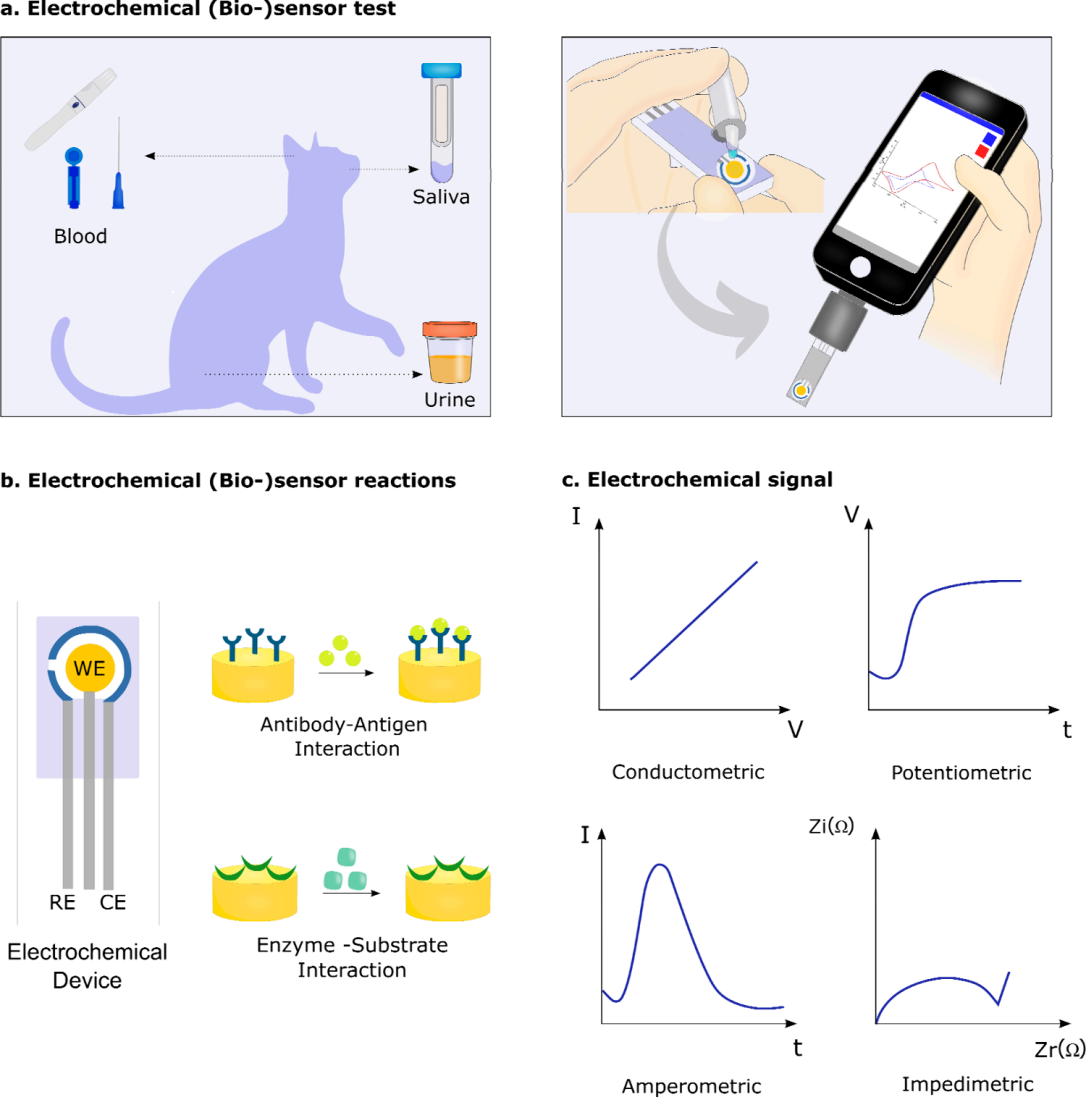
Schematic representation of electrochemical (bio)­sensors for POCT: (a) Devices for blood, saliva, and urine analysis. (b) Electrochemical immunosensor and enzymatic biosensor. (c) Typical responses obtained from different electrochemical transducers. Panels (b) and (c) are adapted and reproduced with permission from ref 131. Copyright 2020 Elsevier B.V.

The main advantages of electrochemical (bio)­sensors as POCT devices for health monitoring and diagnostics include high sensitivity and low LoD, fast and quantitative responses, miniaturization, low cost, and ease of use.^52^ Like LFTs, characteristics such as affordability, simplicity, and rapid response make electrochemical (bio)­sensors ideal for routine monitoring and preventive care. In particular, their high sensitivity, low LoD, and quantitative responses make them powerful tools for health monitoring beyond simple disease screening, providing accurate biomarker concentration measurements even at the early stages of disease progression or in asymptomatic cases. However, limitations such as the need for regular calibration due to variability in samples from different animals, biofouling at the sensor surface, sensor-to-sensor reproducibility issues, and response to interfering species, remain as some of the obstacles to the widespread use of electrochemical devices for pet POCT. We believe these challenges can be addressed through rational engineering of materials, sensor design, chemical functionalization strategies, and machine learning (ML) for enhance data analysis.

### Materials and Strategies for Electrochemical (Bio)­Sensors

Electrode materials and their fabrication methods are key factors governing the sensitivity, LoD, response time, and cost of electrochemical POCT devices. Electrodes can be metallic (e.g., Au) or carbonaceous (e.g., graphene) and processed on diverse surfaces, such as plastic, paper, glass or SiO_2_ wafers, in different dimensions and configurations.^54^

Optical lithography and laser ablation (Figure 6a,b) are main techniques used for patterning metallic electrodes,^55^ while solution-processing is typically employed to fabricate disposable screen-printed electrodes (SPEs)^56^ based on carbonaceous materials and metallic nanoparticles (Figure 6c). 3D printing also represents an interesting strategy for producing low-cost electrodes (Figure 6d). Recently, laser-induced graphene (LIG) has been utilized to produce highly conductive, solvent-free graphene electrodes from different carbonaceous sources.^57^ All these techniques are compatible with industrial-scale manufacturing of POCT devices.

**Figure 6:**
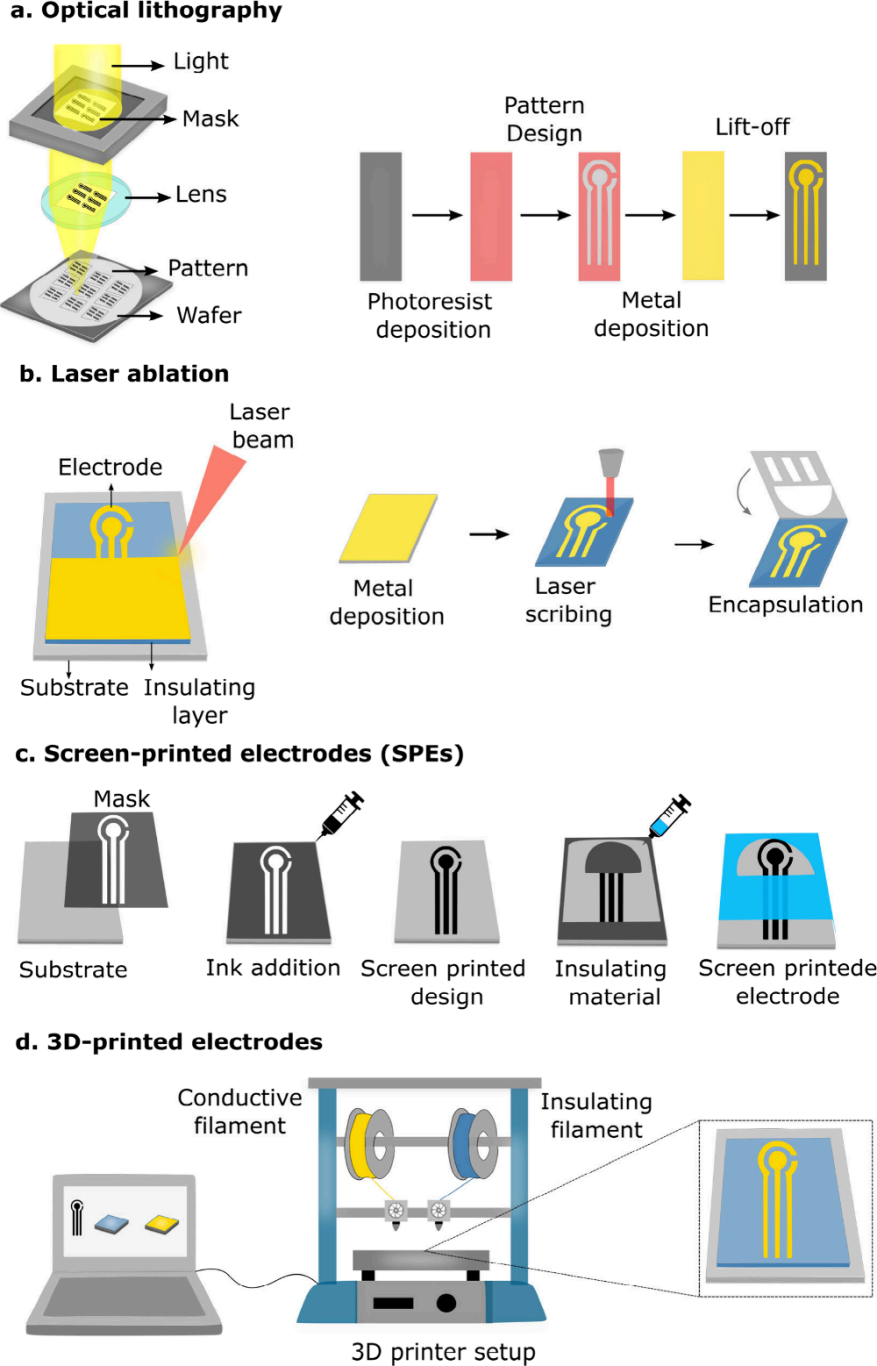
Fabrication strategies for electrochemical devices: (a) optical lithography, (b) laser ablation, (c) screen-printing, and (d) 3D-printing of electrodes.

Some of the properties that are essential for electrodes in POCT devices include high electrical conductivity (10^5^ – 10^7^ S/m), mechanical robustness, and thermal, chemical, and electrochemical stability. In particular, WE and CEs should exhibit high surface area and a wide operational voltage window, while REs must provide a stable and reproducible reference potential.^58^ While many of these properties depend on the materials and fabrication methods used, others can be introduced by exploiting the unique properties of nanomaterials (NMs).^59^

NMs can provide high surface area and chemical versatility for immobilizing (bio)­receptors on WE, enabling analyte selectivity. They can accelerate electron transfer kinetics at the WE surface, improving the device electrochemical response.^56^ NMs can also provide resistance to corrosion and chemical stability to electrodes. The use of NMs in electrochemical (bio)­sensors is a vast field, with extensive literature detailing a variety of applications and functionalization strategies. Readers interested in this topic are encouraged to consult dedicated review articles.^60,61^

As discussed for LFTs, the selection of bioreceptors plays a critical role in the performance of sensing technologies.

Abs are one of the most used biorecognition elements in electrochemical (bio)­sensors due to their inherent high affinity.^62^ Alternatively, enzymes are highly target-specific bioreceptors also widely used in electrochemical biosensors.^63^ In particular, oxidoreductase enzymes catalyze reactions that generate or consume electroactive species, making them ideal for amperometry and voltammetry techniques. Finally, molecularly imprinted polymers (MIPs) are synthetic structures designed to mimic to selectively recognize analytes such as proteins,^64^ DNA,^65^ and small molecules.^66^ MIPs form stable complexes through specific noncovalent interactions, offering advantages such as synthetic versatility, reusability, lower cost, and superior stability compared to enzymes and Abs. They are extensively employed in chemical sensors using transducing technologies like voltammetry, amperometry, and impedance spectroscopy.^67^

### Research Initiatives on Pet POCT Technologies

Diverse scientific efforts have been reported in the literature aiming to develop pet POCT technologies. Here we discuss the most recent publications, focusing on applications with potential clinical and technological relevance.

Cordeiro et al.^68^ developed an impedimetric (bio)­sensor to simultaneously detect Abs related to *Trypanosoma cruzi* (anti-T. cruzi) and Leishmania infantum (anti-L. infantum) in canine serum samples (Figure 7). The biosensor utilizes two SPE modified with distinct bioreceptors to recognize the target Abs in real time. The device demonstrated excellent repeatability, stability over 8 weeks (at 4 °C), and 100% accuracy while distinguishing between positive and negative samples. This method can effectively diagnose Leishmaniasis and Chagas disease in dogs, avoiding common false-positive results. However, biomarker quantification in serum is still a challenge.

**Figure 7:**
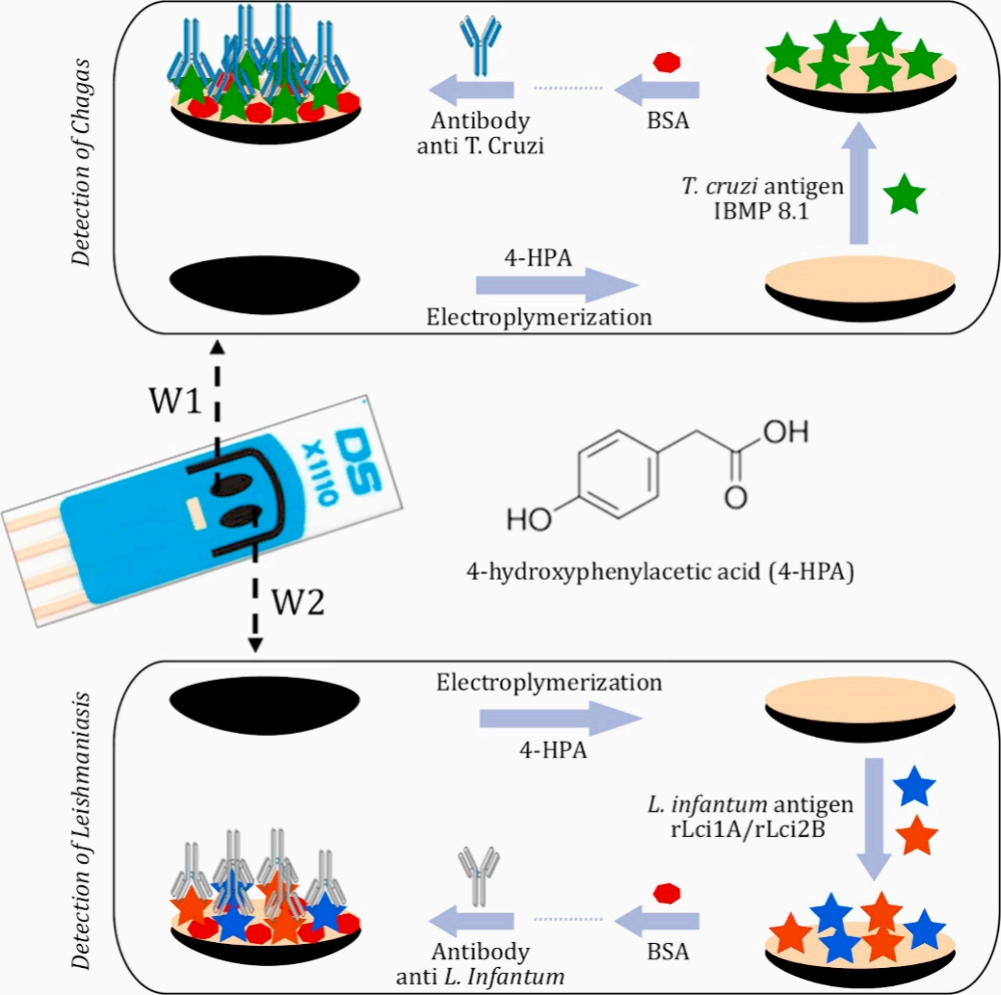
Electrochemical (bio)­sensor for simultaneous detection of *Trypanosoma cruzi* (anti-T. cruzi) and Leishmania infantum (anti-L. infantum) Abs in dog serum samples. Reproduced with permission from ref 68. Copyright 2020 Elsevier B.V.

Döşkaya et al.^69^ developed a testing device for diagnosing toxoplasmosis in cats, the main transmitter of the *Toxoplasma gondii* parasite to humans. Screening cats can prevent the widespread propagation of disease. The method relies on an LFT using recombinant GRA1 protein (rGRA1) to detect anti-T.gondii Abs in serum samples. The device showed fast response (15 min), using 20 μL of serum samples and 200 μL of running buffer. The test exhibited 90% sensitivity and 100% specificity considering 40 samples (30 positive, 10 negative). Although the method is fast, cheap, and requires small sample volumes, the use of rGRA1 requires complex purification steps, potentially limiting accessibility to other users. Since the device detects Abs in serum, and not in whole blood, the technology is more appropriate for veterinary practice rather than for domestic use.

Microfluidic systems have been developed for POCT of pet diseases, including sample-to-answer platforms. Nguyen et al.^70^ developed a benchtop molecular analyzer and a centrifugal microfluidic device (lab-on-a-disk) for detecting pathogens linked to feline upper respiratory tract diseases (FURTD), the most prevalent illness in cats. The targeted pathogens included Feline herpesvirus 1, Chlamydophila felis, Mycoplasma felis, and Bordetella bronchiseptica. The analyzer operation relies on Loop-mediated Isothermal Amplification (LAMP) and Polymerase chain reaction (PCR) detection methods (Figure 8a). The microfluidic device features a centrifugal disc having a glass filter extraction column to purify nucleic acid and multiple reaction chambers for analyte detection (Figure 8b). This allows automatic sample injections, DNA extraction from oropharyngeal samples, multiplex target gene amplification, and complete diagnostics in 1.5 h. This technology is ideal for advanced pet diagnostics at animal hospitals and veterinary clinics.

**Figure 8:**
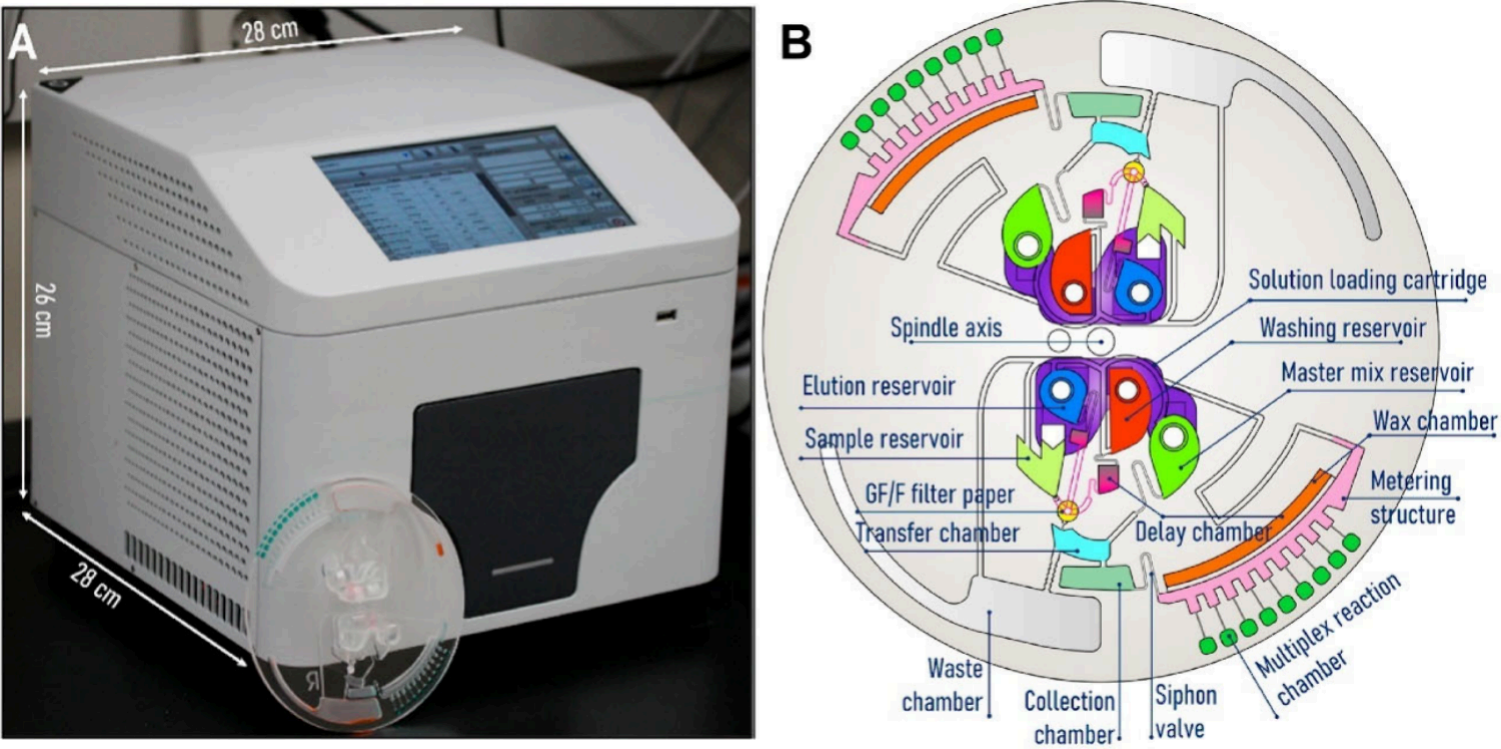
(A) Molecular lab-on-a-disk analyzer. (B) microfluidic disk device. Adapted and reproduced with permission from ref 70. Copyright 2021 Elsevier B.V.

White blood cell (WBC) count is an important method in veterinary medicine for obtaining information about the animal immune system and related diseases. The standard method is based on automated laboratory analyzers based on flow cytometry and light scattering or impedance detection, followed by microscopy blood smear count (Figure 9a). However, this method is difficult to miniaturize. To overcome this limitation, Barroso et al.^71^ developed a POCT hemogram analyzer for WBC count in canine blood using a visible-near-infrared spectroscopy portable device (Figure 9b). They compared traditional chemometric analysis with self-learning artificial intelligence (AI) algorithms, incorporating data augmentation to enhance sensor accuracy.^71^ The AI outperformed chemometrics, achieving a correlation of 0.8478 and a standard error of 6.92 × 10^9^ cells/L, with a mean absolute percentage error of 25.37%. Recently, they reported WBC counting in feline blood samples employing the same POCT technology and AI-based method.^72^

**Figure 9:**
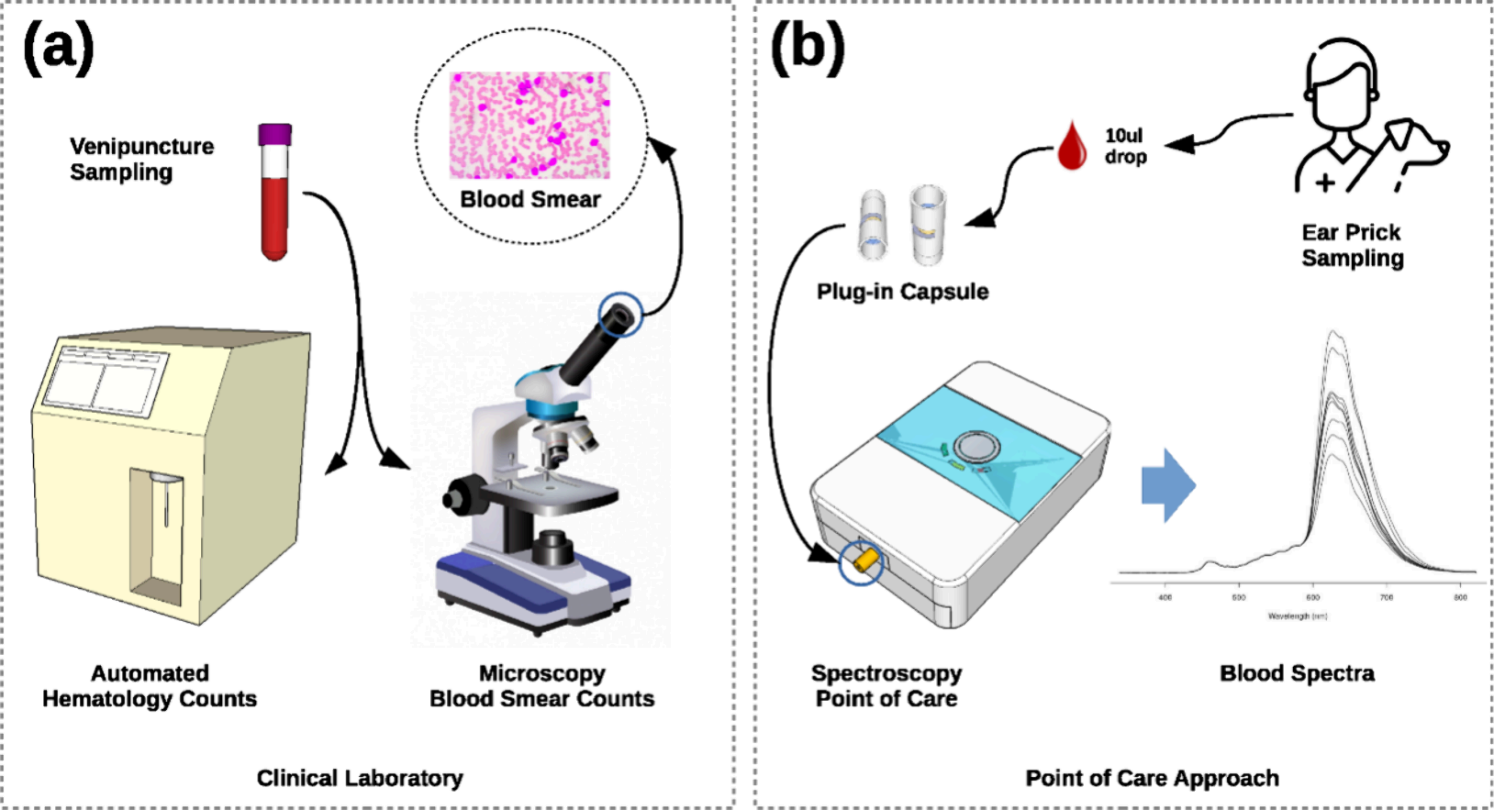
White blood cell (WBC) count methods: (a) conventional and automated laboratory analyzer and manual blood smear counts using a microscope and (b) POCT spectroscopic device from a single blood drop. Reproduced from ref 71 Available under a CC-BY 4.0 license. Copyright 2022 with permission from authors: Barroso T. G., et al.

Another innovative approach for POCT of pets is the work of J.F. Giarola et al.^73^ who developed a multitarget surface plasmon resonance (SPR) biosensor for COVID-19 diagnosis in dogs, cats, and hamsters (Figure 10). The biosensor detects total SARS-CoV-2 Abs (IgG and IgM) in serum. Gold chips are functionalized with the viral antigens (N and S proteins), which bind to the target Abs producing a SPR wavelength displacement. Results are obtained in less than 15 min using only 20 μL of sample, reaching a LoD of 49.6 ng/mL. The method showed 100% sensitivity and 71.4% specificity, well suited for POCT of domestic animals.

**Figure 10:**
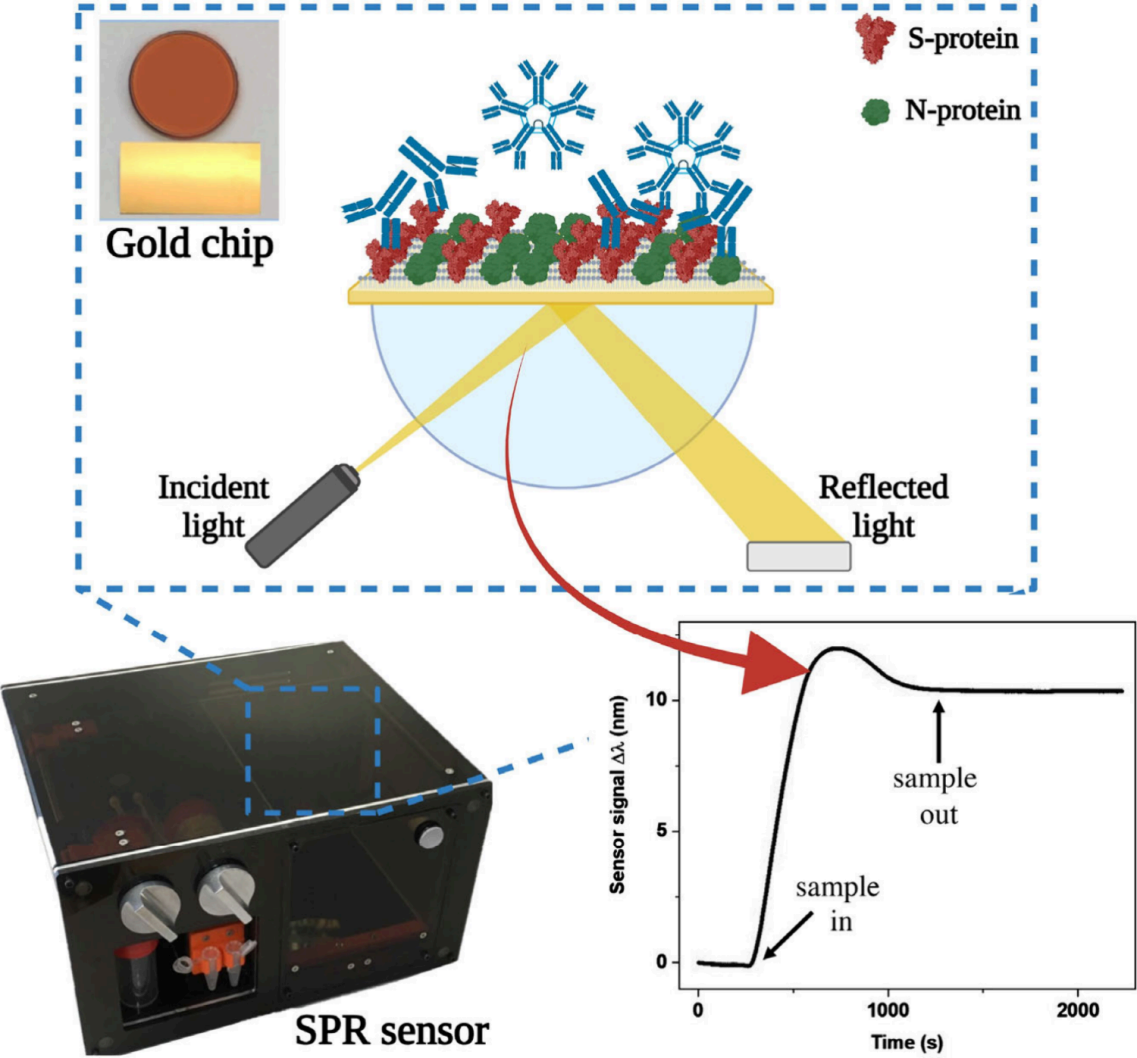
Schematic representation of the SPR POCT device featuring a plasmonic gold chip and real-time tracking of wavelength shifts for sample. The biosensor was employed to detect SARS-CoV-2 Abs in pet serum samples. Reproduced and adapted from ref 73. Available under a CC BY-NC-ND 4.0 license. Copyright 2024 Giarola, J. F., et al.

### Commercial Pet POCT Devices

In addition to scientific research, diverse pet POCT devices have already reached the market. Commercial technologies include blood glucose monitors, urine test strips, and POCTs for a variety of specific diseases. Some distinguished examples are illustrated and discussed below.

Blood glucose monitors provide immediate and accurate results for diabetes diagnosis in companion animals.^74^ Several glucose meters have been designed specifically for pets. These include the brands AlphaTrak 3, iPet Pro, Advocate PetTest, VetMate, and VQ Pet H, to name a few. Most devices are calibrated for dogs and cats, with species selection required during testing. Blood samples are typically collected using a lancing device from specific sites (Figure 11a, step 1–2), such as the ear vein or the paw pad in cats and dogs, the leg callus or the inner lip (for dogs only). A drop of blood (ca. 0.3 μL) is dispensed on a test strip connected to the glucose meter (Figure 11a, step 3). Glucose levels from 1.1 to 41.67 mmol/L can be detected in adult cats and dogs within 5 −10 s with elevated accuracy (>95%). Some technologies permit data to be saved automatically and synced with an app, allowing owners to track information over the long term.

**Figure 11:**
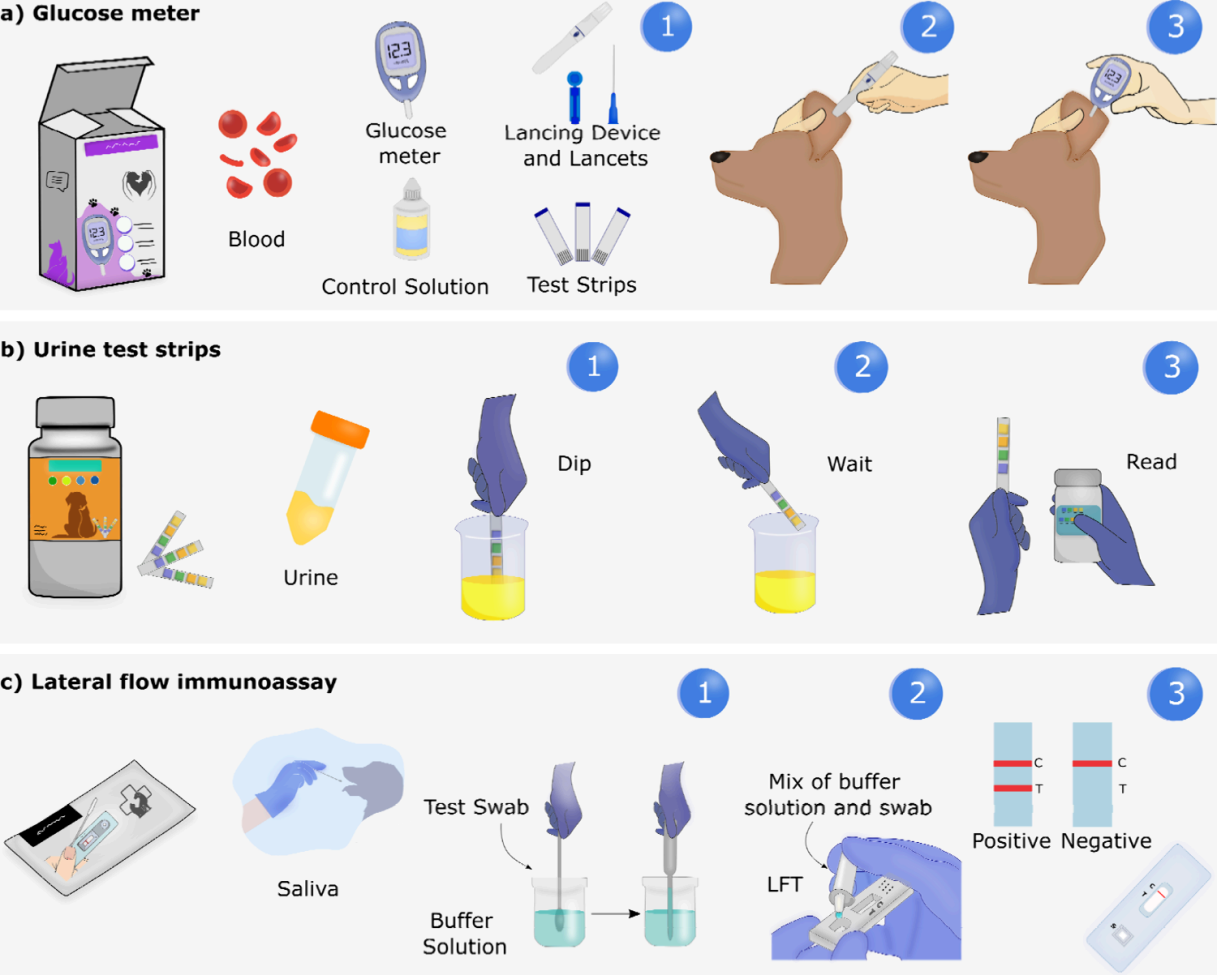
Schematic representation of common commercial POCTs for pet diagnostics and health monitoring. (a) Pet blood glucose monitoring: (1, 2) blood collection using a lancing device and (3) glucose detection. (b) Test strips for urine analysis: (1) immersion of the sensor strip in urine, (2) reaction between the analyte and receptors within the device, and (3) color change based on the analyte concentration. (c) LFT for detecting biomarkers in animal saliva: (1) saliva collection using a test swab, (2) addition of the sample dilution in buffer and addition to the LFT device, and (3) results displayed on the device after the incubation period.

Urine test strips are one of the most used POCT technologies for monitoring the urinary health of pets. The reagent strips contain chemical substances that interact with specific components in the urine, causing a color change depending on the concentration of the analytes. They are designed to simultaneously detect a number of urine parameters and substances, e.g., glucose, nitrite, blood, pH, ketone, leukocytes, etc., providing information about kidney and liver function and diseases (Figure 11b). The strips are immersed in the animal’s urine for a brief period (Figure 13b, steps 1–2), and the results are compared on a color scale to assess the concentration of the measured substance (Figure 11b, step 3). POCT urine strips can be found in the market under different brands for testing the urine of cats and dogs.

The saliva urea test strip Kidney-Chek by SN Biomedical Inc. is a rapid, noninvasive POCT for detecting chronic kidney disease (CKD) in dogs and cats. CDK affects 1 in 10 dogs and 1 in 3 cats 10 years or older, being a silent disease for both animals. Kidney-Chek is a semiquantitative test that measures saliva urea concentration, indirectly reflecting serum urea, for concentrations ranging from 3 to 17 mmol/L.^75^ The test is recommended for screening for azotemia, the accumulation of nitrogenous products in the blood, found in pets with stage 2 CKD. Test strips are rubbed in the animal’s gums for 5–10 s, and results are available after ca. 2 min by direct comparison using color scoring.

Commercial LFTs have been developed for detecting several biomarkers and related diseases, such as canine parvovirus, coronavirus, and Giardia antigen from canine feces, adenovirus antigen from conjunctiva and nasal fluid of dogs, canine Dirofilaria immitis, Ehrlichia canis, Leishmania infantum, and canine brucellosis Abs from dog blood or plasma; feline herpesvirus and calicivirus antigens in conjunctival fluid, heartworm antigen and Abs from feline blood or serum, among others.^76^ Depending on the target and sample type, tests can display results from 5 to 20 min.

One example of LFT commercialized by JCMED for detecting canine distemper, a highly contagious viral disease in dogs (Figure 11c). The Canine Distemper Virus (CDV) test kit can detect viral antigens using two Abs in a sandwich assay from samples such as saliva, plasma/serum, ocular and conjunctival secretions, nasal fluid, and urine from dogs. Saliva samples are collected using a swab, placed into the assay diluent tube, and mixed for approximately 10 s (Figure 11c, step 1). Subsequently, the diluted sample is placed in the sample inlet and the result awaited in 5–10 min (Figure 11c, steps 2–3). LFTs have also been developed for detecting feline immunodeficiency virus (FIV) in cats. Two commercially available POCT kits, Witness and Anigen Rapid, have been shown to accurately distinguish between FIV-vaccinated and FIV-infected cats, offering a viable alternative to PCR testing.^77^ Most LFTs are qualitatively only to screen for the presence of antigens or Abs. The POCT for pet diagnostics market is still in its infancy with respect to technologies for human health. Thus, one may expect accelerated growth and several opportunities in the near future.

## Perspectives

POCTs are essential tools for rapid health monitoring of companion animals, helping owners and veterinarians to prevent, predict, and treat diseases. Although scientific research and commercialization of pet POCT devices have been developed in recent years, investments and advances in this area are significantly smaller compared to those for human health. In the following, we summarize the main perspectives in pet POCT, hoping to stimulate further investigations and investments in the field.

### Adapting Existing Commercial Technologies

Adapting validated human POCT technologies can reduce overall development costs and time, facilitating regulatory approval. For example, glucose meters could be adapted since the target analyte (viz. glucose molecule) is also a marker of diabetes in cats and dogs. However, this would require considering the compositional differences between human and pet blood in terms of glucose concentration, red blood cell count, hematocrit levels, etc., that can impact the glucose readings. Animal clinical trials must be performed considering the blood differences between cats and dogs, and respective breeds. Recalibration of the sensor and validation by standard methods are necessary to ensure the meter can meet the sensitivity and accuracy needed. Finally, partial adaptation of existing technologies is also a possibility, for example, by using commercial glucose meters with test strips designed specifically for animals. However, we advise that not all technologies can be easily adapted since human and pet biomarkers can be different for the same health condition (e.g., pregnancy). Adapting existing POCT technologies can also be an effective measure to quickly respond to emerging diseases and outbreaks.

### Development of New POCT Devices and Applications

When biomarkers are different for humans and pets or existing technologies possess limited performance, developing completely new POCT devices can be a solution. To this end, researchers can follow the ASSURED criteria (Affordable, Sensitive, Specific, User-Friendly, Rapid, Equipment-free, and Delivered) established by the World Health Organization (WHO) for human POCT technologies. Affordability can be achieved by adopting industrial-compatible methods for large-scale production of devices (e.g., printing techniques). Modern technologies will demand materials and processing techniques that can meet sustainability goals for responsible development. High sensitivity is very important to detect subtle quantities of the target analyte, reducing false negatives and increasing reliability. To this end, new transducers can be developed based on optimized materials and device architectures, or even on completely new physical or chemical transducing phenomena (e.g., quantum electron transfer rate).^78^ Researchers must also pursue the optimization of different device key performance indicators (KPIs), such as accuracy, LoD, response time, shelf life, etc. Thus, specificity is also key to avoiding false-positive results. Specific (bio)­receptors for pet diseases can be introduced into device platforms to detect the target pet disease (Tables 1 and 2). POCTs must have an intuitive design, require simple and minimal sample preparation, and rely on automated data recording and analysis to deliver clear and fast results.

Future advancements in POCTs should also aim to incorporate new and improved functionalities. Particularly, multiplexing is desirable for achieving simultaneous detection of multiple analytes from a single sample, offering a comprehensive assessment of the pet’s health necessary for individualized diagnostics and treatment. However, several challenges remain from the sensor manufacturing and analytical point of view to incorporate effective multiplexing. In this sense, lab-on-a-chip microfluidic platforms offer a powerful solution to address fabrication complexities associated with sample handling and multiple detection sites on a miniaturized chip. AI algorithms can be used to handle a large amount of information during simultaneous sensing of several analytes. This integration enhances accuracy and facilitates individualized diagnostics by correlating multiple biomarkers.

### Wearable and Implantable Sensing Technologies

Wearable and implantable sensors represent a frontier in POCT, enabling continuous, real-time health monitoring over extended periods. Although applications are expanding for human health, technologies for pets are much scarcer. Wearables for pets are mostly physical sensors (e.g., motion sensors, heartbeat sensors, respiratory rate detectors, etc.) rather than chemical sensors and biosensors. They are typically integrated into collars and vests and rely on radar and acoustic technologies. In humans, wearable chemical sensors and biosensors exploit direct access to sweat and interstitial fluid using skin contact devices or microneedle patches. However, cats and dogs sweat only through specific areas (e.g., paw pad) and their dense fur obstructs direct skin contact, which requires shaving the area to accommodate the sensor. The natural movement of animals and their unpredictable behaviors add further complexity to the development of wearables. Although some studies have explored the use of human-designed wearable sensors for pets,^79–81^ to the best of as knowledge no commercial technologies exclusive for pets are currently available.

Implantable POCT devices offer an interesting alternative to wearable sensors for continuous, real-time monitoring of animal physiology.^82^ Unlike wearables, they bypass challenges related to skin contact, animal movement and comfort. They also minimize the need for frequent intervention, like device replacement or recharge, while enabling long-term access to internal physiological information.^83^ Additionally, implantable sensors can take advantage of the well-established and widespread use of implantable identification microchips by integrating advanced chemical sensing and biosensing technologies for more comprehensive diagnostics.

The development of implantable POCT (bio)­sensors faces several challenges that hinder their widespread commercialization, both in veterinary and human medicine. Implantable (bio)­sensor often suffer from performance and lifetime issues, including (i) premature device decomposition, (ii) detachment or desorption from the target site, (iii) electrical failure or short-circuiting, (iv) inactivation or loss of bioreceptor activity, (v) fibrotic capsulation due to inflammation or foreign body response, and (vi) biofouling – the accumulation of macromolecules or microorganisms on the device surface.^83^ Addressing these challenges requires innovative design strategies, extensive use of advanced materials, and biocompatibility studies.^84^ Given that animals are frequently used as models to evaluate implantable (bio)­sensors, developing devices specifically tailored for pet diagnostics represents an act of care and commitment to animal well-being. A recent and comprehensive review paper on implantable sensors for in vivo monitoring of animal physiology can be found elsewhere.^85^

### Breath Sensors

Another important branch of pet POCT technology is breath sensing for detecting exhaled volatile organic compounds (VOCs) biomarkers. Specific VOC levels in exhaled breath are associated with different diseases. For example, acetone is a known biomarker for diabetes in both humans and pets (dogs and cats), where individuals show acetone levels 2–5 times higher than healthy ones.^86,87^ Other relevant breath biomarkers include hydrogen sulfide (H_2_S) for gastrointestinal diseases, ammonia (NH_3_) for kidney disease, nitric oxide (NO) for respiratory diseases, in addition to viruses and bacteria.^86^ Breath monitoring can be used to screen and monitor disease progression, helping with early diagnosis, prevention, and treatment. However, the complexity of breath samples (thousands of components) and the low concentration of biomarkers (typically sub ppb to ppm) present challenges for sensor sensitivity and data analysis. Common sensing technologies include optical sensors, chemiresistors, and electrochemical biosensors.^86^ Breath sensors are noninvasive and allow online real-time monitoring using wireless communication. Compared to human health research, POCT for breath analysis in pets remains underdeveloped. Potential research directions include (i) adapting human-based technologies to expand biomarker databases for animal health and (ii) developing pet-specific POCT devices that integrate advanced materials, multiplexed detection, and ML for enhanced analysis.

### Biomarker Discovery

Research on the discovery of biomarkers plays an important role in human and veterinary diagnostics, allowing one to identify new molecular indicators of diseases and responses to treatments. Biomarker discovery typically involves omics technologies (genomics, proteomics, metabolomics), bioinformatics and ML to analyze large data sets from patients. Once identified, potential biomarkers are then analytically and clinically validated; from this point, new POCT devices can be designed to detect the target biomarker at clinically relevant concentrations in real samples.

Compared to the research in human medicine, biomarker discovery in veterinary is less developed. The difference between animal and human physiology prevents the direct application of human biomarkers to pets. The diversity of breeds within the same specie further complicates the identification of universal pet biomarkers. This is because biomarker concentration ranges may differ across species and breeds. This genetic variability demands personalized and highly specialized approaches to ensure the clinical applicability of discovered biomarkers. AI-driven models can help process vast amounts of omics and clinical data, identifying correlations that might be overlooked using traditional methods.^88,89^

Although biomarkers are being actively investigated in veterinary medicine, only a limited number have undergone full clinical validation. A major barrier to translating biomarker research into clinical application is the lack of regulatory standards for analytical methodology.^90^ This gap hinders the commercialization of POCT technologies for newly identified animal biomarkers. Addressing these challenges will require continuous investment and collaboration between academia and industry. The interdisciplinary nature of this field presents a unique opportunity to advance POCT for pets and establish new diagnostic standards.

## Final Remarks

The success of POCT technologies for human health underscores their potential to revolutionize animal healthcare, although several challenges remain. Advancing in this field requires expanding and diversifying research efforts, driving product development, and fostering new business opportunities. Establishing a multilateral dialogue between veterinarians, materials scientists, biotechnologists, and data scientists, is a crucial step toward addressing key challenges efficiently and effectively.

Currently, most reported POCT devices have not reached full technological maturity (viz. Technology Readiness Level, TRL),^91^ and commercial sensors face challenges similar to those in human health care, such as high occurrence of false positive and negative results. Research initiatives should focus on developing high-performing POCTs that match the sensitivity and accuracy of standard laboratory methods in veterinary practice. Regulatory agencies play a critical role in evaluating POCTs, ensuring their safety, efficiency, and compliance with veterinary clinical standards. Additionally, researchers must align scientific development with market demands.

Adopting digital technologies will be decisive for individualized diagnostics, via long-term tracking of pet health status and information sharing between veterinarians, animal hospitals, and pet owners. This has significant implications for primary care and prevention, early disease detection, and treatments tailored to the needs of each animal. Moreover, synchronizing these data could empower health authorities to monitor potential zoonotic hotspots and detect early indicators of emerging epidemics, enabling timely and targeted interventions. We are convinced that advancing pet POCT is fundamental to achieving a practical and integrated human-environmental-animal One Health.

## Vocabulary

Companion Animals (Pets): Domesticated species, specifically dogs and cats, that have evolved a preference for humans, form strong bonds, communicate with and understand humans, and once socialized remain voluntarily with them.
Chemical Sensor: A device that converts chemical information, ranging from the concentration of a specific sample component to total composition analysis, into an analytically useful signal.
Biosensor: A device that uses specific biochemical reactions mediated by isolated enzymes, immunosystems, tissues, organelles, or whole cells to detect chemical compounds usually by electrical, thermal or optical signals.
Point of Care Test: Medical or veterinary diagnostic test conducted at or near the patient, delivering rapid and reliable results to identify and monitor diseases, accelerating treatment decisions.
Lateral Flow Test: A portable, low-cost, rapid diagnostic device, known as immunochromatographic test, in which a small volume of patient fluid (blood, urine, or saliva) is applied to the device and migrates along a membrane via capillary action, interacting with immobilized reagents to produce a visual or instrumental signal.
Electrochemical Biosensors: A self-contained integrated device capable of providing specific quantitative or semiquantitative analytical information using a biological recognition element, which is retained in direct spatial contact with an electrochemical transduction element.
Biomarker: A measurable indicator of normal or abnormal biological processes, disease conditions, or responses to treatment, typically a biomolecule or a molecule of biological origin.
Multiplexing: Capability of a sensor or biosensor to simultaneously detect and measure multiple analytes or biomarkers from a single sample.
One Health: An integrated and unifying approach that aims to sustainably balance and optimize the health of individuals, animals, and ecosystems.
